# Deposition Dosages of Three Cromolyn Forms by Cascade Impactor

**DOI:** 10.1155/2017/1892725

**Published:** 2017-04-02

**Authors:** Norihide Murayama, Kei Asai, Kikuno Murayama, Chihiro Kitatsuji, Satoru Doi

**Affiliations:** ^1^Murayama Pediatrics, 3-2-33 Nagayoshi-Nagahara-Higashi, Hirano-ku, Osaka-shi 547-0013, Japan; ^2^Omron Healthcare, Co., Ltd., 53 Kunotsubo, Terado-cho, Hyuga-shi, Kyoto 617-0002, Japan; ^3^Nagasaki University, 1-12-4 Sakamoto, Nagasaki-shi, Nagasaki 852-8523, Japan; ^4^Osaka Prefectural Medical Center for Respiratory and Allergic Diseases, 3-7-1 Habikino, Habikino-shi, Osaka 583-8588, Japan

## Abstract

Among inhaled asthma therapies, the present study aimed to identify factors for selecting the type of inhalation therapy for asthma. Three methods are used to deliver inhaled cromoglycate, and the airway deposition rate was evaluated using a cascade impactor with 3 dosage forms: dry powder (DP), pressurized metered dose inhaler (pMDI), and solution (jet- and mesh-types). The percentage of particles with diameters of 2–6 *μ*m was 17.0% for the capsule, 51.8% for pMDI, 49.0% for jet-type NE-C28, and 40.4% for mesh-type NE-U22. The amounts of drug deposited in the bronchi were based on the particle distribution of the various dosage forms: 3.4 mg for the capsule, 1.0 mg for pMDI, 9.8 mg for one solution (jet-type NE-C28), and 8.1 mg for the other solution (mesh-type NE-U22). Jet-type or mesh-type electric nebulizers delivered 2-3 times more of the drug than capsules, and, compared with pMDI, 8-9 times more of the drug was deposited in the bronchi/bronchioles. Electric nebulizers are considered the best method. This study suggests that the size of particles deposited at sites of obstruction is larger than previously reported, and no obstruction of small airways occurs (<2 mm).

## 1. Introduction

The site and amount of deposition of an inhaled antiasthma drug in the airway are very important in the evaluation of treatment for asthma. However, since drugs for asthma are designed and manufactured according to each company's policy, their particle size varies, and studies comparing them have been limited.

In 1996 [1], we conducted experiments based on the assumption that the efficacy of inhaled drugs must be evaluated on the basis of the assessment of the amounts deposited in the airway. Cromoglycate (Intal; Astellas Pharma Inc., Tokyo, Japan), when inhaled, is absorbed via the airway and is rapidly excreted into urine without being metabolized in the liver. Using this characteristic, we compared 3 types of electric nebulizer (mesh-type, jet-type, and glass aspirator). It was the highest with the mesh-type, being 2-fold higher than that by the jet-type and 4-fold higher than that by the glass sucker, and the differences were greater than expected. But these methods could not explain the deposition site (bronchial generation) of cromoglycate. Although these differences are considered to be caused by various factors, including the residual quantity of the solution drug, air flow velocity, and particle size, we focused on the particle size, which varied most widely among the 3 inhalation types. With cascade impactor we explain about deposition site and rate from particle size spectrums.

### 1.1. Cascade Impactor [2]

In this present study we used Marple Personal Cascade Impactor 298. Specifications of this device are as follows: cut-off points of stage impactor 1, 2, 3, 4, 5, 6, 7, and 8 are 21.3, 14.8, 9.8, 6.0, 3.5, 1.55, 0.93, and 0.52 micrometers, respectively. Setting of flow rate is 2 liters per minute nominal in 0.5 to 5 L/minuets max. Range of 1 to 3 L/minuets is recommended. Construction is precision-machined aluminum and impactor stages are nickel plated.

**Figure 1:**
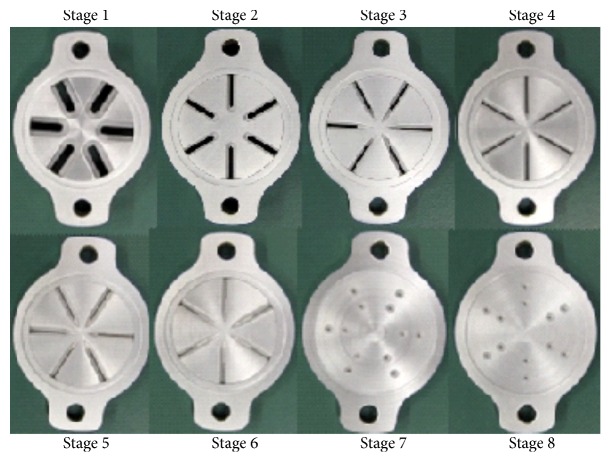
A Marple-type cascade impactor. Each compartment of the impactor (stages 1 to 8) and the slit size (mean value) of each stage. The slit size is indicated by width and length (stages 1 to 6) and diameter (stages 7 to 8). In a cascade impactor, the flow velocity through the slits increases in more distal stages. The situation is the complete opposite in the human airway.

The cascade impactor is a device to capture an aerosol using inertial collision to study the particle size and properties of aerosol in the atmosphere [3, 4]. The Marple-type cascade impactor (Figure 1, Table 1) consisted of 8 compartments called stages. Stages 1 to 6 have slits, arranged with 30° horizontal staggers from one stage to the next. This mimics the sequential 30° horizontal shifts of the bronchial bifurcations in the human body. Stages 7 and 8 have holes rather than slits. As the stages advance (at more distal sites of the airway model), the total cross-sectional area of the slits is considered to decrease and the flow velocity to increase. This is the opposite of the bronchial bifurcations, in which the total cross-sectional area increases at more distal sites [5], and the cross-sectional area is maximized at the terminal alveolar generation, where the flow velocity becomes zero.

Theoretically, the cut-off diameter of particles that are deposited by inertial collision is determined at each stage, and particles of 2–6 micrometers in diameter are considered to be deposited in the bronchi/bronchioles [6]. It is thought that particles are deposited on a cascade impactor primarily by inertia, and actual deposition by diffusion or sedimentation in the terminal airway of the lung of the living body is not taken into consideration. The development of airway tree models closer to the living lung is awaited.

## 2. Materials and Methods

### 2.1. Method of Test for Particle Sizing

The methods used to measure aerosol size were based on those defined in European Standard EN13544-1. Low flow cascade (Marple Series 298X) was loaded with cut GF/A filters and connected to a T-piece. The cascade impactor for measuring particle size from jet-type nebulizer NE-C28, mesh-type NE-U22, DP, and MDI was set up so that 2 L/min of air was pulled through the impactor. And the flow rate from all of mesh-type NE-U22, jet-type NE-C28, and MDI was set as 13 L/min, and the flow rate from DP was set as 60 L/min. 2 mL of disodium cromoglycate (DSCG) was set to 2 kinds of nebulizers and the nebulizers were allowed to continue for 3 minutes, 20 puffs (1 mg/a puff) for MDI, and a capsular (20 mg) form DP has emitted.

All of disodium cromoglycates (DSCG) trapped on to the GF/A filters were assayed by the HPLC (SHIMADZU LC-10ADVP (Shimadzu Corp., Tokyo Japan)) under the following conditions.

1. Column: Shim-pack VP-ODS 150 × 4.6 mm,
2. Mobile phase: methanol : phosphate buffer 6 : 4,
3. Retention time of 3 minutes.

### 2.2. Evaluation of the Airway Deposition Rate Using a Cascade Impactor

**Figure 2:**
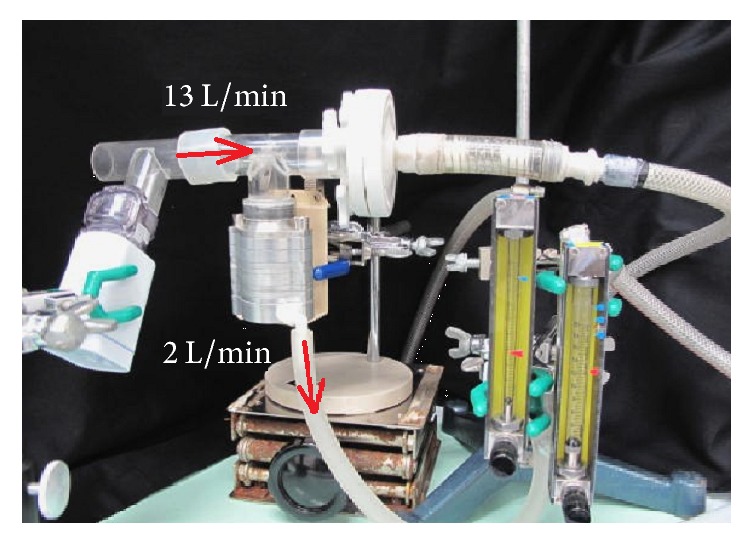
Measurement of particle size distribution with a mesh-type nebulizer NE-U22 using a cascade impactor.

The airway deposition rate was evaluated according to the particle size spectrum using a cascade impactor. Presently, inhaled drugs for asthma are available as DP, MDI, and solution for both adults and children. Since cromoglycate is marketed in all 3 dosage forms, comparisons among dosage forms of the same drug are possible. The site and amount of deposition of inhaled antiasthma drugs are important in evaluating the efficacy of treatments for asthma, but reports of evaluations using the same setups and conditions and different dosage forms or devices have been limited. Therefore, the amounts of cromoglycate deposited in each stage of the Marple-type cascade impactor, which is regarded as a standard in Europe, were evaluated by delivering the drug in 3 dosage forms (capsule (DP), pMDI, and inhalant solution (with the jet-type NE-C28 or the mesh-type NE-U22 nebulizer)) (Figure 2), the particle size distribution was studied, and the deposition rate in the bronchi was evaluated.

## 3. Results and Discussion

### 3.1. Results

**Figure 3:**
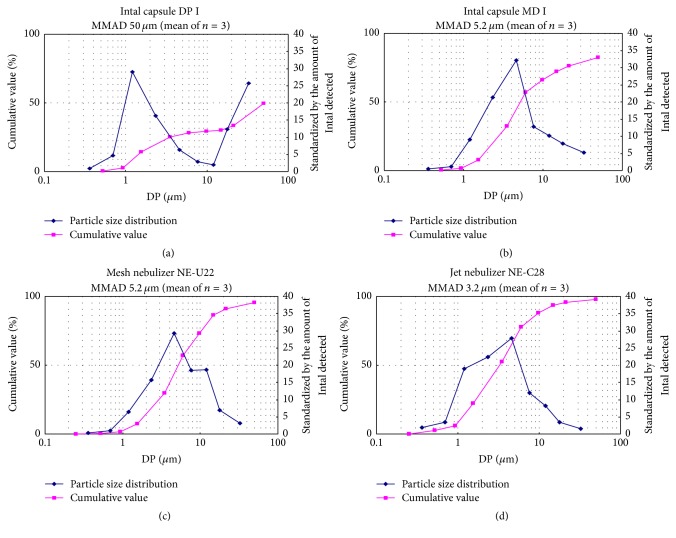
Results of analysis using a cascade impactor. Particle size distribution and cumulative values in 4 dosage forms: (a) Intal capsule (DP), (b) Intal MDI, (c) one solution inhalant (a mesh-type nebulizer NE-U22), and (d) the other solution inhalant (a jet-type nebulizer NE-C28) are shown in blue and pink, respectively. The data points represent the means of three independent experiments.

The massive medium aerodynamic diameter (MMAD) of particle size distribution was 50.0 *μ*m for the capsule (DP), 5.2 *μ*m for pMDI, 3.2 *μ*m for one solution inhalant (jet-type NE-C28), and 5.2 *μ*m for the other solution (mesh-type NE-U22) (Figure 3). It was within 2–6 *μ*m, and a particle size range deposited in the bronchi, in all dosage forms except the capsule preparation. The distribution pattern was bimodal for the capsule (dry powder) but unimodal for the other dosage forms. The percentage of particles within 2–6 *μ*m in diameter, which are considered to be deposited in the bronchi, was 17.0% for the capsule, 51.8% for pMDI, 49.0% for one solution (jet-type NE-C28), and 40.4% for the other solution (mesh-type NE-U22) (Table 2). Since the amount of drug delivered by 1-time use is 2 mg (2 puffs) with pMDI, but 20 mg with a capsule or solution, the amount deposited in the bronchi based on the particle distribution of various dosage forms was 3.4 for the capsule, 1.0 mg for pMDI, 9.8 mg for one solution (jet-type NE-C28), and 8.1 mg for the other solution (mesh-type NE-U22). Thus, by delivering a solution using an electric nebulizer, the amount of drug deposited in the bronchi/bronchioles is estimated to be 2-3 times that delivered by the capsule and 8-9 times that delivered by pMDI.

### 3.2. Discussion

**Figure 4:**
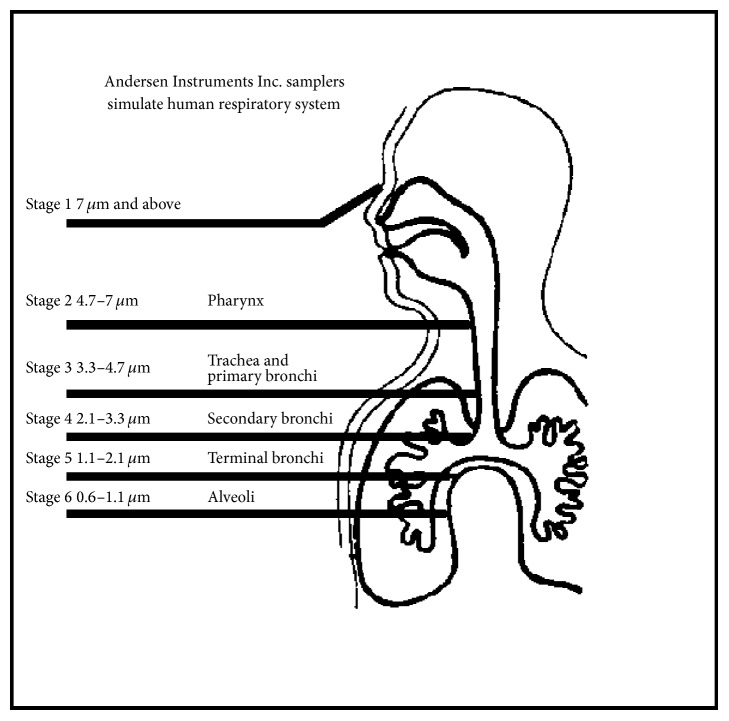
Excerpt from the instructions for use of the Marple-type cascade impactor.

In evaluating bronchial deposition, the appropriate particle size varies markedly depending on which of the 1st to 16th branches of the airway (including bronchioles) are targeted for the treatment. Particles of 2–6 *μ*m in diameter, thought to be deposited in the bronchi by analysis using the cascade impactor, are targeted to relatively proximal bronchi (Figure 4). However, the sites of bronchi that are affected by asthma rather than the sites of particle deposition should be discussed first.

Weibel's respiratory tree model [7], first reported in 1963, was adopted in the ICRP text book of the respiratory tract and is still cited in papers published in the 2000s [8, 9]. The model is very interesting. The airway bifurcates repeatedly, and the airways from the 1st to the 4th generations are called bronchi, those from the 5th to the 16th generations are bronchioles, those from the 17th to the 19th are respiratory bronchioles, and those from the 20th to the 23rd generations are alveolar ducts and sacs. Since the luminal wall of the 17th and more distal generations is composed of the alveolar wall, the airways from the 1st to the 16th generations can be called bronchioles. Of these airways, those less than 2 mm in diameter, from the 8th to the 16th generations, are called small airways (peripheral bronchi). The bronchi initially bifurcate by reducing the internal diameter at a rate of $1/\sqrt{2}$, but the rate of tapering decreases gradually in more distal areas. Therefore, the more distal the airways, the greater the total cross-sectional area. Compared with the tracheal cross-sectional area (2.54 cm^2^), the total cross-sectional area shows 0.98-, 2.7-, 11.3-, and 70.8-fold increases in the 4th, 8th, 11th, and 16th generations, respectively. The authors consider the airways of the 1st to 7th generations, which bifurcate serially at a rate near $1/\sqrt{2}$, to be the sites of physiological stenosis in the airway. Furthermore, as the airway resistance decreases progressively in distal areas, inflammation is observed in the entire bronchial tree in bronchial asthma [10], and inflammation of the distal airways is not reflected in respiratory function [11], we consider that inflammation of this part of physiologic stenosis to be the primary pathology of asthma. The decreases in V50 and V25 shown by spirometry are interpreted as signs of small airway obstruction, but there has been no report on which generations are obstructed. The authors consider that decreases in V50 and V25 indicate obstruction of the site of physiologic stenosis in relatively large airways (narrowed further due to inflammation caused by asthma), which collapse during expiration.

Evaluation of the urinary excretion of cromoglycate indicated that the amount of drug deposited in the airway shows several-fold variation depending on the delivery method (type of electric nebulizer), even when an identical amount of the same drug is administered. This method has also recently been used to evaluate the amount of airway deposition [12]. Analysis using a cascade impactor showed that mesh-type and jet-type electric nebulizers have advantages compared with pMDI or capsules for the inhalation of cromoglycate. According to the instructions for the Marple-type cascade impactor, particles of 2–6 *μ*m in diameter are considered to be targeted to relatively large bronchi. On evaluation using Weibel's human airway model, spirometry indicated obstruction of relatively large bronchi [13] but no obstruction of small airways. Therefore, an aerosol with a relatively large particle size is considered necessary for the treatment of asthma.

HFA-BDP (Qvar; Sumitomo Dainippon Pharma Co., Ltd., Osaka, Japan), which first appeared in the literature in 1998 [14], attracted attention as an aerosol with a very small particle size (MMAD: 1.1 *μ*m). Its lung deposition rate was reported to be 50% or higher, but as the peripheries of the lungs are delineated on technetium deposition imaging, a large part of the drug aerosol particles is considered to be deposited in the alveoli. Thus, their distribution is considered to deviate from the treatment target area, which is assumed to be the 1st–7th generations of the airway tree. However, treatment using an ultrafine aerosol may be more effective for asthma complicated by eosinophilic pneumonia with lesions in the alveolar region, asthma showing pulmonary remodeling, and asthma complicated by COPD. However, the HFA used in pMDIs, although not an ozone-depleting substance, has a greenhouse effect 1,300-fold stronger than CO_2_, and the early development of a medium that can be used as a substitute for it is awaited. Presently, HFA is used in all pMDI-type drugs for asthma.

## 4. Conclusions

There are three kinds (dry powder, pMDI, and solution) of dosage forms for cromoglycate inhalation asthma therapy. Aerosol particle size from 2 to 6 *μ*m is considered to reach affected regions of the bronchi with a cascade impactor. We have different amounts by different forms of cromoglycate from 2 to 6 *μ*m particle size. Compared to dry powder of cromoglycate, pMDI and solutions have three-fold 2–6 *μ*m percentages. The one-time dosages of cromoglycate are far different among three forms. The one-time dosages of pMDI, solution, and dry powder are 2, 20, and 20 mg, respectively. From the one-time use dosage and deposition rate by cascade impactor, a solution with a jet or mesh nebulizer is the most advantageous dosage form. We consider that from the anatomical data this present steroid pMDI's particle size is much smaller compared to our present data.

## Figures and Tables

**Table 1:** Sizes of cascade impactor slits.

Stage number	Width (mm)	Length (mm)
1	2.60	9.51
2	1.43	9.48
3	0.84	8.91
4	0.43	9.52
5	0.27	9.03
6	0.17	4.64

	Diameter (mm)	

7	0.45	
8	0.34	

**Table 2:** Results of the study.

	Capsule	pMDI	Jet-type nebulizer C-28	Mesh-type nebulizer NE-U22
MMAD (*μ*m)	50.0	5.2	3.2	5.2
Distribution pattern	Bimodal	Unimodal	Unimodal	Unimodal
Percentage of particles 2–6 *μ*m	17.0%	51.8%	49.0%	40.4%
Standard dose per one-time use (mg)	20	2	20	20
Amount deposited in the bronchi by administration of treatment dose (mg)	3.4	1.0	9.8	8.1

MMAD: massive medium aerodynamic diameter, pMDI: pressured metered dose inhaler.

## Abbreviations

COPD: Chronic obstructive pulmonary disease
DP: Dry powder
MMAD: Massive medium aerodynamic diameter
pMDI: Pressurized metered dose inhaler.
